# Human Endogenous Retrovirus Expression Is Upregulated in the Breast Cancer Microenvironment of HIV Infected Women: A Pilot Study

**DOI:** 10.3389/fonc.2020.553983

**Published:** 2020-10-22

**Authors:** Gislaine Curty, Greta A. Beckerle, Luis P. Iñiguez, Robert L. Furler, Pedro S. de Carvalho, Jez L. Marston, Stephane Champiat, Jonas J. Heymann, Christopher E. Ormsby, Gustavo Reyes-Terán, Marcelo A. Soares, Douglas F. Nixon, Matthew L. Bendall, Fabio E. Leal, Miguel de Mulder Rougvie

**Affiliations:** ^1^Oncovirology Program, Instituto Nacional de Câncer, Rio de Janeiro, Brazil; ^2^Division of Infectious Diseases, Department of Medicine, Weill Cornell Medicine, New York, NY, United States; ^3^Drug Development Department (DITEP), Gustave Roussy, Paris-Saclay University, Villejuif, France; ^4^Department of Pathology and Laboratory Medicine, Weill Cornell Medicine, New York, NY, United States; ^5^Center for Research in Infectious Diseases (CIENI), National Institute of Respiratory Diseases (INER), Mexico City, Mexico

**Keywords:** breast cancer, HIV, human endogenous retrovirus, retrotranscriptome, microenvironment, formalin-fixed paraffin-embedded, telescope software, breast cancer oncogenes

## Abstract

In people living with HIV (PLWH), chronic inflammation can lead to cancer initiation and progression, besides driving a dysregulated and diminished immune responsiveness. HIV infection also leads to increased transcription of Human Endogenous Retroviruses (HERVs), which could increase an inflammatory environment and create a tumor growth suppressive environment with high expression of pro-inflammatory cytokines. In order to determine the impact of HIV infection to HERV expression on the breast cancer microenvironment, we sequenced total RNA from formalin-fixed paraffin-embedded (FFPE) breast cancer samples of women HIV-negative and HIV-positive for transcriptome and retrotranscriptome analyses. We performed RNA extraction from FFPE samples, library preparation and total RNA sequencing (RNA-seq). The RNA-seq analysis shows 185 differentially expressed genes: 181 host genes (178 upregulated and three downregulated) and four upregulated HERV transcripts in HIV-positive samples. We also explored the impact of HERV expression in its neighboring breast cancer development genes (*BRCA1, CCND1, NBS1*/*NBN, RAD50, KRAS, PI3K*/*PIK3CA*) and in long non-coding RNA expression (AC060780.1, also known as RP11-242D8.1). We found a significant positive association of HERV expression with *RAD50* and with AC060780.1, which suggest a possible role of HERV in regulating breast cancer genes from PLWH with breast cancer. In addition, we found immune system, extracellular matrix organization and metabolic signaling genes upregulated in HIV-positive breast cancer. In conclusion, our findings provide evidence of transcriptional and retrotranscriptional changes in breast cancer from PLWH compared to non-HIV breast cancer, including dysregulation of HERVs, suggesting an indirect effect of the virus on the breast cancer microenvironment.

## Introduction

The majority of mammary tumors in mice are caused by the mouse mammary tumor virus (MMTV) (1), and the suggestion that a retrovirus related to MMTV might be involved in breast cancer in humans is one of the longest running controversies in human retrovirology (2, 3). Early studies of the breast cancer cell line T47D showed retroviral particles which were responsive to estradiol (4–8). These were later identified as human endogenous retrovirus (HERV)-K envelope transcripts, and were found both in cell lines and in breast cancer tissues (9, 10). HERVs are “fossil” retrovirus sequences remnant in the human genome, which originated millions of years ago from retrovirus germline cell infections. They are transmitted vertically, but they do not show infective capabilities in humans (11–13). They comprise ~8% of the human genome (14).

In women with breast cancer, HERV-derived material has been found in peripheral blood (15, 16). The consequences of changes in HERV expression include translation of some HERV proteins and stimulation of anti-HERV immune responses (17–19). A chimeric antigen receptor (CAR) specific for HERV-K env protein was effective in reducing tumor burden in a mouse model (20). The corollary is that HERV-K expression may be related to oncogenesis, and that HERV-K Env proteins appear to play an important role in tumorigenesis and metastasis (21). A recent study showed that the endogenous retrovirus-derived long noncoding RNA (lncRNA) TROJAN promotes triple-negative breast cancer progression via ZMYND8 degradation (22). Thus, expression of HERVs in breast cancer may relate to pathogenesis, but also to generation of anti-HERV immunity with protective potential.

In HIV infection, we, and others, have found that HERV expression is changed in CD4+ cells (23–41), and that it is partially mediated by HIV Vif (28). Although no bystander specific HERV activation has been observed, interestingly, people living with HIV (PLWH) have higher levels of HERV-K RNA expression, which is negatively associated with the level of T cell activation (28, 31).

No study has yet addressed the impact of HIV mediated HERV transcription in breast cancer. Since breast cancer cell lines and tissues appear to express HERVs, and the local microenvironment of breast cancer contains potentially HIV infected-CD4+ cells, there is the possibility of direct or indirect interaction between HERVs dysregulated by HIV and HERVs dysregulated in breast cancer. We undertook this retrospective pilot analysis of HERV expression from archived formalin-fixed, paraffin-embedded (FFPE) tissue samples in women who had breast cancer with or without HIV infection.

## Materials and Methods

### Sample Collection

FFPE blocks of breast tissue from 10 women with invasive ductal carcinoma (IDCA) of the female mammary gland (four HIV-negative: six HIV-positive) registered from 2003 to 2014 at the Brazilian National Cancer Institute (INCA), were used in this study.

The study was approved by the local Institutional Review Board, as well as by the Brazilian National Ethics Committee. All subjects had signed informed consent forms in accordance with the Declaration of Helsinki.

### RNA Extraction

FFPE tissue blocks were sectioned into 3 μm thick curls and placed in 2 mL Safe-Lock tubes (Eppendorf, cat. no. 022600044), with the first three 3 μm sections discarded. No more than ten sample curls were placed in each Eppendorf tube, ~30 μm worth of material, according to manufacturer's instructions of a maximum 40 μm processing limit. Samples were then deparaffinized in 320 μL Deparaffinization Solution (Qiagen, cat. no. 19093) at 56°C for 3 min, then allowed to cool at room temperature.

RNA extraction and purification from FFPE tissue sections were carried out using the RNeasy FFPE Kit (Qiagen, cat. no. 73504). The RNeasy MinElute spin columns were incubated for 5 min with RNase-free water before centrifugation. Samples were eluted in 20 μL RNase-free water. A DNase treatment was added using HL-dsDNase kit (ArticZymes, cat. no. 70800-201 250U), with additive MgCl_2_ (ThermoFisher Scientific, cat. no. AB0359) and DTT, Molecular Grade (Promega, cat. no. P117A). RNA samples were DNase-treated at room temperature for 15 min, followed by a 55°C 10-min incubation to complete enzyme activity. RNA was stored at −80°C.

### RNA Quantification and Quality Assessment

RNA was assessed for quantity on a Qubit™ 2.0 Fluorometer using the RNA Broad Range Assay Kit (Invitrogen, cat. no. Q32855). RNA was assessed for quality and integrity on an Agilent Bioanalyzer 2100 unit using Agilent RNA 6000 Nano Series II Kit (Agilent Technologies, cat. no. 5067-1511).

### Library Preparation and Quality Control

RNA samples were prepared with Ovation® Human FFPE RNA-Seq Library Systems (Nugen, cat. no. 0340, 0341). Clean-up steps, including magnetic bead purifications, were performed with freshly made 70% EtOH from 100% proof Absolute Ethanol (Fisher BioReagents™, cat. no. BP2818-500), nuclease-free water (Ambion®, cat. no. AM9939), and DynaMag™-96 Side magnetic plates (Invitrogen, cat. no. 12331D). As a rule, each library was amplified for 18 PCR cycles. Amplified library products were routinely checked for quantity and purity before pooling for Next Generation Sequencing. Final products were assessed on an Agilent Bioanalyzer 2100 unit using the High Sensitivity DNA Kit (Agilent Technologies, cat. no. 5067-4626). Final products were also assessed using KAPA Library Quantification Kit (Illumina) and/or ROX Low qPCR Mix (Roche, cat. no. 07960336001). If samples passed quality control inspection, each library prep was diluted at 4 nM using 10 mM Tris-HCl, 0.1% Tween-20, pH 8.5 (Teknova, cat. no. T7724). Libraries were pooled in 8 sample sets, and sequenced in a paired-end mode, Mid-Output platform on an Illumina NextSeq 2000 Sequencing System.

### Bioinformatics

BCL2FastQ2 Conversion Software (version 2.20, Illumina Inc.) was used to demultiplex data and convert the sequencing files to reads in FASTQ format. Paired-end reads were trimmed for adaptors with Trimmomatic (42) and filtered by quality (phred ≥ 30) and length (≥ 35). Filtered paired-end reads were then aligned to the reference human genome (hg38) using Bowtie2 (43). The Bowtie2 output was used as input for Telescope software (44) to define and quantify retrotransposon elements (HERV and LINE-1) using annotations, previously described and available at https://github.com/mlbendall/telescope_annotation_db/tree/master/builds.

In addition, we mapped all reads to the human genome (hg38) with STAR (45). The output was then used for gene quantification using Htseq-count (46) with Gencode version 31. Telescope and Htseq-count table outputs were merged and genes or retrotransposon elements which were present in less than two samples were removed. The processed output was then used to calculate HERVs and genes differentially expressed genes in breast cancer from HIV-positive vs. HIV-negative patients using DESeq2, Wald-test (47).

Volcano plots were drawn with the Bioconductor EnhancedVolcano (https://github.com/kevinblighe/EnhancedVolcano). HERVs and host genes with adjusted *p*-value <0.05 and absolute(log2FoldChange) > 1.0 were considered differentially expressed genes (DEGs) and results were shown using pheatmap and ggplot R packages. HERV localization in the human genome was visualized with integrative genomics viewer (IGV) software (Broad Institute, Cambridge, MA) using Gencode version 31 and HERV annotations as previously described (41). We also analyzed nearby HERVs to genes associated with breast cancer development (Supplementary Table 1) (48–50). The HERV localization was defined and HERV and nearby gene expression results were shown in graphs using ggplot R packages.

Gene set enrichment analysis (GSEA) was performed with WebGestal (available at http://webgestalt.org/) using Reactome as functional pathway database (51). We also analyzed the differentially immune gene expression, including CD4 and CD8 (CD8A and CD8B) mRNA in the samples to check T-cell-derived material in transcriptome data. The graphs were constructed using the ggplot R and the adjusted *p*-value was calculated from DEseq2 analysis performed using the Wald-test.

All data generated in the study has been deposited in GEO under the accession number GSE149156.

### Immunohistochemistry

Immunohistochemical (IHC) was done using a standard protocol for FFPE tissues. Breast tumor biopsies from six HIV-positive and four HIV-negative women were sectioned into 10 μm slices and then deparaffinized, rehydrated, and subjected to antigen retrieval with 10 mM sodium citrate buffer and heat for 10 min. Staining was done using antibodies for four differentially expressed genes PROM1/CD133 (CST D2V8Q XP® Rabbit mAb #64326, 1:400 dilution), LAMB3 (Abcam antibody ab97765, 1:400 dilution), SLC6A4 (ThermoFisher polyclonal antibody PA5-50624, 1:100 dilution), and MRPS12 (ThermoFisher polyclonal antibody 15225-1-AP, 1:50 dilution). Detection of the signal was done using SignalStain Boost Detection Reagent (Cell Signaling Technology) and brightfield images were taken using a Keyence BZ-X810 microscope.

## Results

**Figure 1:**
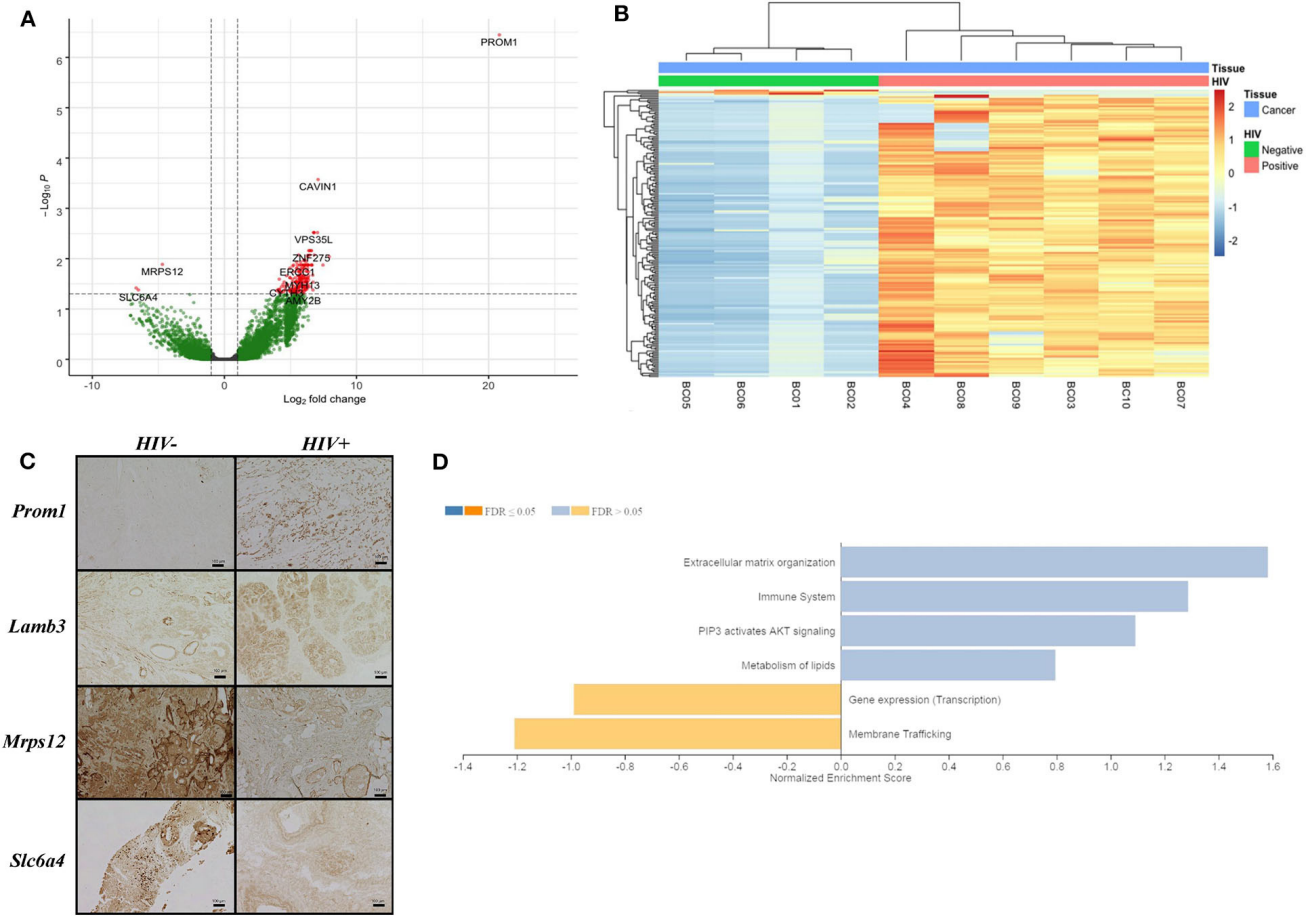
Differentially expressed genes in breast cancer from HIV patients. **(A)** Volcano plot shows differentially expressed HERV and host genes, up-regulated (right side) and down-regulated (left side) in breast cancer HIV-positive samples. Threshold lines to adjusted *p*-value <0.05 (x-axis) and log2FoldChange > 1.0 (y-axis) are shown. Green and red colors indicate significant and non-significant genes, respectively, found in the differential gene expression analysis. **(B)** Heatmap plot demonstrates 181 differentially expressed genes in the horizontal dendogram to breast cancer samples (labeled in blue) from HIV-positive (labeled in pink) and HIV-negative (labeled in green). Unsupervised clustering for rows and columns was performed using Euclidean distances and a complete linkage method. **(C)** Immunohistochemical analysis of four host proteins (PROM1, LAMB3, SLC6A4, and MRPS12) in HIV-negative and HIV-positive breast cancer tissues. **(D)** GSEA illustrates cellular pathway gene enrichment in HIV-positive samples. False discovery rate (FDR) shows up- and down-regulated pathways in light orange and blue, respectively, for *q*-value > 0.05, while *q*-value <0.05 is represented in dark orange and blue.

We analyzed RNA-seq data of breast tissue from women with IDCA HIV-positive and -negative breast cancer. Samples from both HIV-positive and -negative groups were predominantly of luminal subtype and of advanced stage at diagnosis (Table 1). The one case of triple-negative IDCA was not of medullary subtype, and there was no morphologically apparent increase in tumor-infiltrating lymphocytes in any of the samples. No patient received neo-adjuvant therapy before resection. It is important to note that, although gene expression data was available, IDCA subtyping was performed using immunohistochemical analysis (IHC) of estrogen receptor and progesterone receptor and a combination of IHC and *in situ* hybridization analysis, as necessary. The transcriptomic analyses showed no significant difference in expression of *ESR1, ESR2, PGR*, or *ERBB2* by RNA-Seq analysis. We found a total of 181 DEGs when comparing both groups, HIV-positive and HIV-negative breast cancer [FDR <0.05, absolute(log2FoldChange) > 1.0; Figures 1A,B]. One hundred and seventy eight of those genes were upregulated and three were downregulated in HIV-positive breast cancer (Supplementary Table 2). *PROM1, LAMB3, OSR1*, and *CAVIN1* were the most upregulated host genes in HIV-positive women with a long2foldchange of 20.8, 7.9, 7.5, and 7.1, respectively (Supplementary Table 2). In contrast, SLC5A11, SLC6A4 and MRPS12 were the most downregulated in HIV-positive breast cancer with a long2foldchange of −6.7, 6.5 and −4.7, respectively (Supplementary Table 2). To biologically validate these findings, we performed IHC assay to detect the expression of four proteins (PROM1, LAMB3, SLC6A4, and MRPS12) generated from those genes found to be differentially expressed in HIV-positive breast cancer samples (Figure 1C). PROM1 and LAMB3 showed lower protein levels in HIV-negative breast cancer tissues compared in those found in HIV-positive breast cancer tissues. Conversely, protein levels of Mrps12 and Slc6a4 were increased in HIV-negative tissues compared to HIV-positive tissues. Thus, these data corroborate with transcriptome results (Supplementary Table 2).

**Table 1:** Demographic and pathologic data of breast cancer samples from HIV-negative and HIV-positive women.

**Demographic data**	**HIV-negative**	**HIV-positive**
	**% (*N*/Total)**	**% (*N*/Total)**
**Median age (min–max)**	47.5 (45–56)	46 (45–51)
**Cancer molecular subtype**
Luminal	75 (3/4)	66.7 (4/6)
HER2+	25 (1/4)	0 (0/6)
Triple-negative	0 (0/4)	16.7 (1/6)
Not available	0 (0/4)	16.7 (1/6)
**Cancer staging**
2A	25 (1/4)	16.7 (1/6)
2B	25 (1/4)	16.7 (1/6)
3A	0 (0/4)	16.7 (1/6)
3B	25 (1/4)	0 (0/6)
3C	25 (1/4)	16.7 (1/6)
4	0 (0/4)	33.3 (2/6)

Furthermore, the results showed enrichment of extracellular matrix organization, immune system, PI3K/AKT signaling and lipid metabolism pathways, all upregulated in HIV-positive samples, but at non-significant levels (Figure 1D and Supplementary Table 3). Transcription machinery and membrane trafficking pathways were found to be downregulated in HIV-positive samples (Figure 1D and Supplementary Table 3).

**Figure 2:**
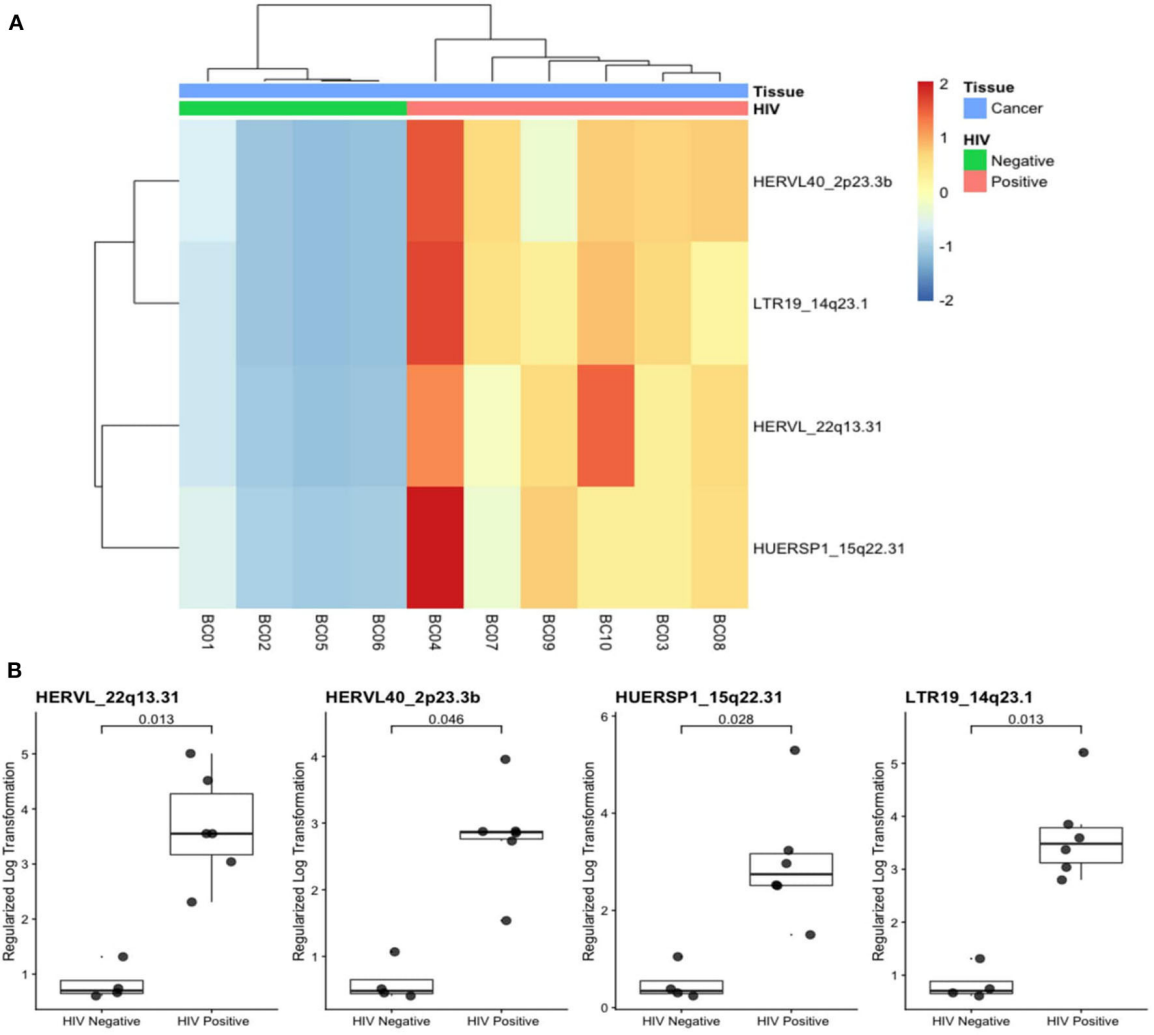
Differentially expressed HERV transcripts in breast cancer from HIV-positive patients. **(A)** Heatmap plot shows the four differentially expressed HERVs in horizontal dendogram to breast cancer samples (labeled in blue) from HIV-positive (pink) and HIV negative (green) are annotated. **(B)** Expression of the four HERV transcripts with adjusted *p*-values are shown.

**Figure 3:**
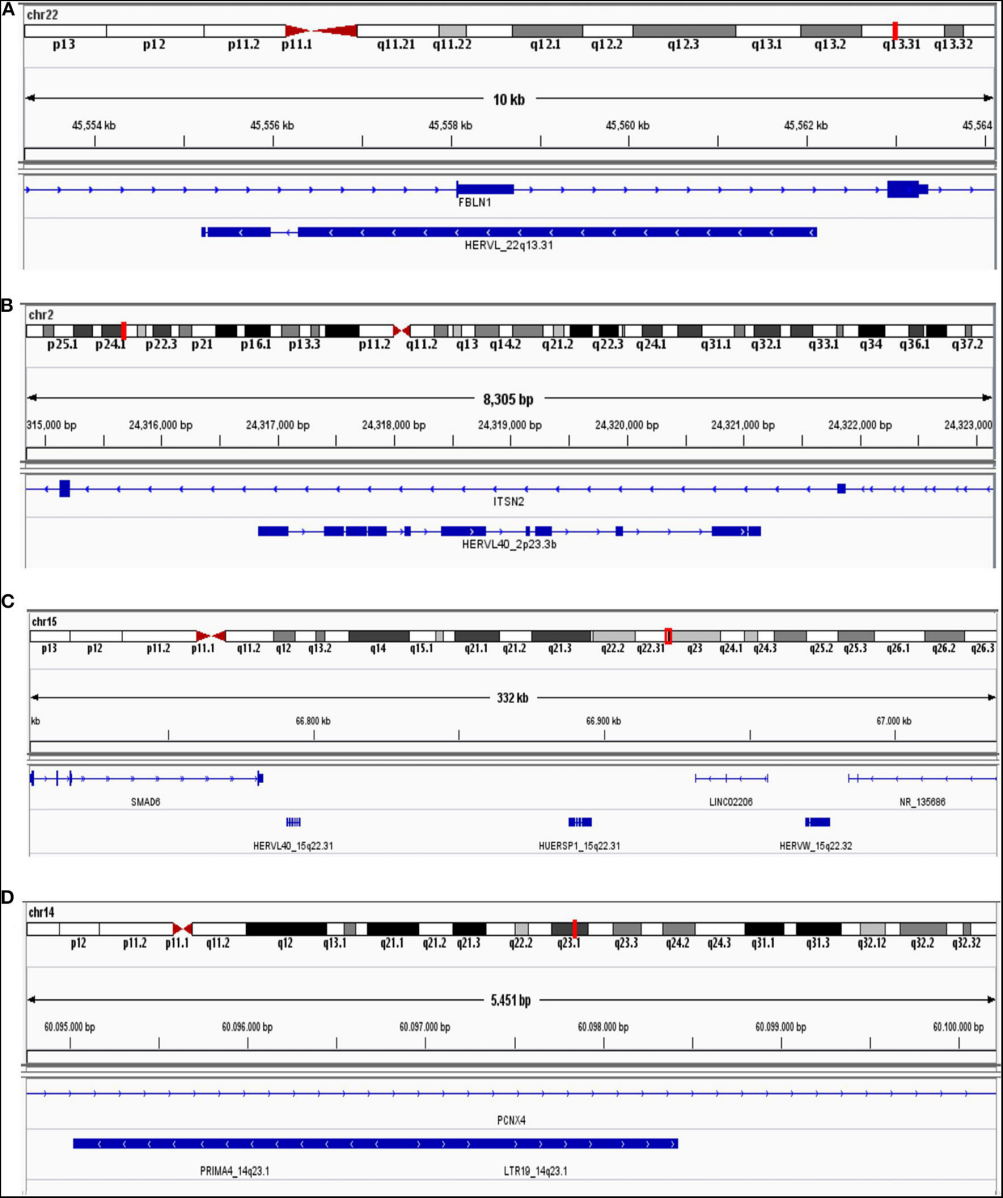
Genome localization from differentially expressed HERV genes. HERVL_22q13.31 **(A)**, HERVL40_2p23.3b **(B)**, HUERSP1_15q22.31 **(C)**, and LTR19_14q33.1 **(D)** are shown in human genome (hg38). Chromosome number and region, beside exon (blue square) and intron (blue line) are illustrated.

Additionally, we quantified locus-specific HERV expression at 14,968 genomic loci using Telescope, a software developed for solving multimapping reads, and performed a combined differential expression analysis with all annotated genes. Four HERV transcripts (HERVL_22q13.31, HERVL40_2p23.3b, HUERSP1_15q22.31, LTR19_14q23.1) were significantly upregulated in HIV-positive samples (Figure 2). HERVL40_2p33.3b and LTR19_14q33.1 were present in the intronic region of intersectin (ITSN2) and Pecanex 4 (PCNX4) genes, respectively (Figures 3B,D and Supplementary Figures 1B,D), HERVL_22q13.31 localized in an alternative polyadenylation site of Fibulin 1 (FBLN1) gene and HUERSP1_15q22.31 is an intergenic HERV (Figures 3A,C and Supplementary Figures 1A,C). Despite none of these host genes was statistically differentially expressed, we analyzed their expression in association with their nearby HERV expression (Supplementary Figure 1). Interestingly, the HUERSP1_15q22.31 expression was associated with the upstream *SMAD6* long non-coding RNA expression, lnc-SMAD6 (AC110048.2), in HIV-positive samples (*R*^2^ = 0.86, *p*-value = 0.029) (Supplementary Figure 1C). In the same way, the LTR19_14q23.1 and PCNX4 expression also showed a positive association in HIV-positive samples (*R*^2^ = 0.86, *p*-value = 0.027) (Supplementary Figure 1D). In addition, we also analyzed HERVs that were associated, by localization, with breast cancer development genes (*BRCA1, CCND1, ATM, NBS1*/*NBN, RAD50, KRAS, PI3K*/*PIK3CA*) (Supplementary Table 1). Although both HERV and genes in this analysis were not differentially expressed in HIV-positive samples, we found significant association between *RAD50* and its intronic HERV, ERVLE_5q31.1d, expression (*R*^2^ = 0.82, *p*-value = 0.044) (Supplementary Figure 2A). Additionally, the last two exons of the *BRCA1* long non-coding RNA, *lnc-BRCA1* (AC060780.1), are contained in the HARLEQUIN_17q21.31 and therefore the expression of both elements, the lncRNA and the HERV are also correlated (*R*^2^ = 0.96, *p*-value = 0.0019) (Supplementary Figure 2D). These results suggest a possible role of HERVs in the regulation of at least some of the breast cancer development genes.

**Figure 4:**
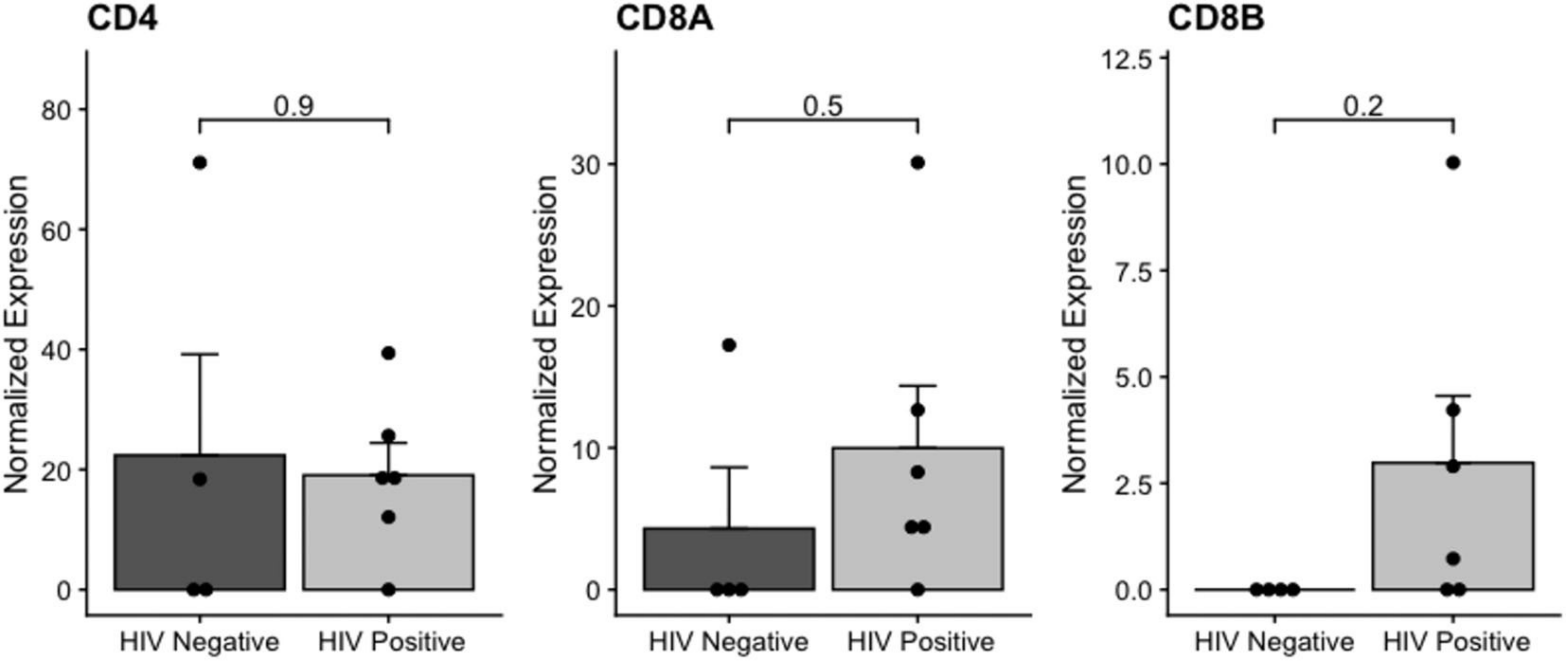
Normalized CD4 and CD8 expression in HIV-positive and HIV-negative from breast cancer samples. The adjusted *p*-value is shown (FDR).

We also analyzed CD4 and CD8 mRNA expression in the samples, as a proxy for the presence of T-cell-derived material in transcriptome data. The CD8 mRNA expression was higher in HIV-positive than in HIV-negative samples (Figure 4), but at non-significant levels. We further analyzed the differential expression of T-cell signature genes (Supplementary Figure 3), and we found the *PIK3IP1* gene upregulated in HIV-positive samples. This pilot study highlighted the importance of additional studies to confirm the increase of tumor-infiltrating lymphocytes and their immune effects on the IDCA microenvironment in HIV-positive patients.

## Discussion

Breast carcinoma is a heterogeneous malignancy originating from the epithelium of the terminal ductal-lobular unit, and it is the most common malignancy in women worldwide (52). Interestingly, women living with HIV (WLWH) have an approximate 40% lower risk for developing this kind of cancer (SIR = 0.63, 95% CI = 0.58–0.68) and have a lower frequency of breast tumors larger than 5 cm (SIR = 0.65, CI 95% = 0.50–0.83) (53). However, the biological mechanism associated with the lower incidence of breast cancer in WLWH is unknown.

In this study, we have described the transcriptome and retrotranscriptome of breast cancer FFPE samples from HIV-positive and HIV-negative women with breast cancer. RNA extracted from FFPE samples may be partially degraded, and performing RNA sequencing from these samples is technically challenging (54–56). However, studies have shown that RNA-seq from FFPE tissues can be used as an alternative for frozen samples, and it can promote advances in clinical retrospective studies, especially in rare types of cancer. The rarity of breast cancer among WLWH poses a major hurdle to prospective analyses of fresh tumor samples, which yields higher-quality sequencing data.

Herein, we found 181 differentially expressed genes and HERVs between HIV-positive and HIV-negative women with breast cancer, with 178 genes upregulated in HIV-positive samples. These data agree with a recent study on HIV-related diffuse large B-cell lymphoma (57), in which digital gene expression profiling and array comparative genomic hybridization from FFPE samples were performed. Diffuse large B-cell lymphoma from HIV infected patients showed differential gene expression, and genes associated with cell cycle progression, DNA replication and DNA damage repair were significantly increased in HIV-positive compared to HIV-negative tumors (57). HIV infection is characterized by persistent inflammation and cell-signaling pathway dysregulation in immune cells (58). Together, these results show that HIV may regulate the host gene expression and impact cellular pathways in addition to inducing HERV expression.

The upregulation of HERV has been implicated in oncogenesis and metastases of breast cancer cells (21). Expression of the HERV-K protein np9 is increased in breast cancer (19) and is considered a viral oncogene due to its association to cellular signaling pathways such as WNT, ERK, Akt and Notch1 (59, 60). HERV and breast cancer associations are frequently based on the analysis of HERV-K expression (9, 16, 19, 61, 62). In this study, we did not find differential HERV-K expression profiles in breast cancer samples from HIV-positive women when compared to samples from HIV-negative women. However, expression of other HERV families have been described in various other types of cancer such as colorectal, stomach and others (63). Studies of HIV/HERV associations also mostly have relied on HERV-K analyses (28, 31, 32, 64).

Furthermore, one critical aspect of HIV pathogenesis is the suppression of the intracellular viral restriction factor APOBEC3 by the HIV protein Vif in CD4 T cells (65). Reduced APOBEC3 activity has been associated with activation of HERV in HIV infection (28, 66). Despite the defined associations between HERV and both HIV and breast cancer, very little is known about the impact of HERV expression in the context of breast cancer in WLHW.

In the current study, we used the Telescope program, a pipeline for locus specific HERV expression identification (44, 67). Telescope is able to identify HERV locus specific expression in RNA-seq data with higher accuracy than other methods (44). We found four overly expressed HERV genes in HIV-positive from breast cancer samples (HERVL_22q13.31, HERVL40_2p23.3b, HUERSP1_15q22.31, LTR19_14q23.1), two of them from HERV-L clades. Interesting, we previously reported HERV-L specific immunity in HIV-1 infection, characterizing a potential novel target for assessment of HIV pathogenesis (27). Moreover, HERV-L shows elevated numbers of somatic single-nucleotide variations in cancer (68), which may impact cancer progression. In addition, HERVs are able to induce carcinogenesis and cancer progression by regulating nearby host genes (12, 69, 70). Herein we reported positive association between expression of some HERVs and their neighbor genes. The HUERSP1_15q22.31 and LTR19_14q23.1, differential expressed in HIV-positive samples, were associated with the *SMAD6* long non-coding RNA and *PCNX4* expression, respectively. In addition, the analysis of HERVs and breast cancer oncogenes also showed a positive association in HIV-positive samples. The ERVLE_5q31.1d and HARLEQUIN_17q21.31 were associated, respectively, with *RAD50* and *BRCA1* long non-coding RNA expression. Mutation in *RAD50* is associated with breast cancer, genome instability and poor survival (71, 72). Additionally, dysregulation of long non-coding RNA expression has been associated with tumorigenesis (73, 74). Together, these data suggest the association between HERV and host gene expression, including oncogenes, which may have a key role in carcinogenesis in breast tissues from HIV-positive persons. HERV expression has also been involved in the aggressiveness and plasticity of cancer cells, showing an important effect in the tumor microenvironment (75). We also found an upregulation of CAVIN1 (caveolae associated protein 1) in HIV-positive breast cancer samples. This protein, which is present in abundance at the cell surface is associated with caveolae formation, and it has been proposed as tumor suppressor protein (76, 77). In contrast, studies have shown proteins of the CAVIN family downregulated in breast cancer (77) from HIV-negative samples. These findings suggest that upregulated CAVIN1 in HIV infection might have an important role in HIV-positive breast cancer suppression. In addition, PROM1 (prominin 1) was found upregulated in HIV-positive breast cancer samples, a gene commonly overexpressed in ovarian, esophageal and liver cancer (78). Its expression is negatively associated with cancer prognosis (78, 79). However, its biological function and role are not well understood in breast cancer.

The breast cancer microenvironment is complex, and there are many interactions between different kind of cells that may impact breast cancer gene expression (80–82). Interestingly, we found a higher but non-significant CD8 mRNA expression in HIV-positive breast cancer compared to HIV-negative, suggesting a higher concentration of tumor-infiltrating lymphocytes in these samples. We also found immune pathway enrichment in HIV-positive breast cancer women according to GSEA. In addition, by analyzing immune T-cell signature genes, we showed *PIK3IP1* upregulated in HIV-positive breast cancer samples. The PIK3P1 protein is a negative regulator of T-cell activation and of antitumor T-cell immunity (83, 84). Interestingly, tumor-infiltrating lymphocytes are able to induce an immune response, and their presence is often associated with a good prognosis across a spectrum of malignancies in HIV-negative patients (85, 86). Our findings may also indicate differential immune response regulation and lymphocyte recruitment in the HIV-related breast cancer microenvironment. Therefore, this pilot report highlights the importance of additional studies of HIV-positive breast cancer patients to evaluate HERV expression effects on breast cancer cells.

Based on our results, the impact of HIV infection in the context of breast cancer in WLWH might rely on a specific HERV expression profile different from the previously HERV-K and breast cancer association seen in HIV-negative women. This pilot study is the first to analyze the impact of HIV infection in breast cancer using transcriptome and retrotranscriptome data and it can serve as basis to further studies. Despite of limited samples number due the rarity of breast cancer among WLWH, our findings suggest that HIV infection might indirectly modulate host and HERV gene expressions in the breast cancer microenvironment. Future studies to better understand the interplay between HIV and HERVs in the breast cancer microenvironment are warranted.

## Data Availability Statement

The datasets presented in this study can be found in online repositories. The names of the repository/repositories and accession number(s) can be found below: the NCBI Gene Expression Omnibus (GSE149156).

## Ethics Statement

The studies involving human participants were reviewed and approved by the local Institutional Review Board, as well as by the Brazilian National Ethics Committee. The patients/participants provided their written informed consent to participate in this study.

## Author Contributions

GC, GB, LI, SC, CO, GR-T, MS, DN, MB, FL, MdM, and RF: conceptualization. GC, GB, LI, PdC, JM, and RF: investigation. GC and DN: writing original draft preparation and writing review and editing. MS, DN, MB, FL, and MdM: supervision. DN: funding acquisition. All authors contributed to the article and approved the submitted version.

## Conflict of Interest

The authors declare that the research was conducted in the absence of any commercial or financial relationships that could be construed as a potential conflict of interest.
