# Supplementary material for: Human Endogenous Retrovirus Expression Is Upregulated in the Breast Cancer Microenvironment of HIV Infected Women: A Pilot Study
Source: Front Oncol. 2020 Oct 22;10:553983. doi: 10.3389/fonc.2020.553983 (PMC7649802; doi:10.3389/fonc.2020.553983)
Supplement: Supplementary file 1 [file Data_Sheet_1.PDF]

Table S1 - List of genes associated with breast cancer development and their neighboring HERVs

Gene	HERV	HERV inserted into gene	HERV inserted Upstream (pb)	HERV inserted downtream (pb)	HERV expression	Gene expression
BRCA1	HARLEQUIN_17q21.31	yes	none	none	yes	yes
AC060780.1	HARLEQUIN_17q21.31	yes	none	none	yes	yes
RAD50	ERVLE_5q31.1d	yes	none	none	yes	yes
CCND1	ERVLE_11q13.3c	none	30690	none	no	yes
NBN	ERV316A3_8q21.3f	yes	none	none	no	no
KRAS	HERVL_12p12.1b	none	56216	none	no	yes
BRCA2	none	none	none	none	none	yes
TP53	none	none	none	none	none	yes
PTEN	none	none	none	none	none	yes
ERBB2	none	none	none	none	none	yes
MYC	none	none	none	none	none	yes
ATM	none	none	none	none	none	no
CHK2	none	none	none	none	none	yes
RAD50	none	none	none	none	none	yes
PALB2	none	none	none	none	none	yes
PI3K	none	none	none	none	none	yes
