# Supplementary material for: Human Endogenous Retrovirus Expression Is Upregulated in the Breast Cancer Microenvironment of HIV Infected Women: A Pilot Study
Source: Front Oncol. 2020 Oct 22;10:553983. doi: 10.3389/fonc.2020.553983 (PMC7649802; doi:10.3389/fonc.2020.553983)
Supplement: Supplementary file 2 [file Data_Sheet_2.PDF]

Table S2- List of differentially expressed host genes in HIV-positive breast cancer

ENSEMBL	Status	SYMBOL	log2FoldChange	adjusted p-value
ENSG00000108576	Down-regulated	SLC6A4	-6.506988885	0.0418605097
ENSG00000128626	Down-regulated	MRPS12	-4.695098235	0.01299700355
ENSG00000158865	Down-regulated	SLC5A11	-6.676065921	0.03818827186
ENSG00000006788	Up-regulated	MYH13	5.911862918	0.02448621319
ENSG00000007062	Up-regulated	PROM1	20.81349982	0.0000003574101447
ENSG00000008256	Up-regulated	CYTH3	4.702955321	0.03472007496
ENSG00000012061	Up-regulated	ERCC1	5.533768825	0.01337110367
ENSG00000012232	Up-regulated	EXTL3	5.795540935	0.01337110367
ENSG00000014919	Up-regulated	COX15	4.526208894	0.02906261826
ENSG00000018408	Up-regulated	WWTR1	4.14552084	0.04578895771
ENSG00000050165	Up-regulated	DKK3	4.156901647	0.04481179636
ENSG00000063587	Up-regulated	ZNF275	6.584245445	0.006920683522
ENSG00000063587	Up-regulated	LOC105373378	6.584245445	0.006920683522
ENSG00000064933	Up-regulated	PMS1	6.127471572	0.03486169293
ENSG00000066629	Up-regulated	EML1	6.03743775	0.03457735026
ENSG00000067798	Up-regulated	NAV3	6.335108607	0.02404314784
ENSG00000070770	Up-regulated	CSNK2A2	6.282665214	0.02404314784
ENSG00000072756	Up-regulated	TRNT1	4.509536811	0.04481179636
ENSG00000075239	Up-regulated	ACAT1	5.382878307	0.0418880173
ENSG00000075399	Up-regulated	VPS9D1	4.911882338	0.04174427651
ENSG00000082014	Up-regulated	SMARCD3	5.353864254	0.02404314784
ENSG00000090612	Up-regulated	ZNF268	5.887184831	0.03606118789
ENSG00000090975	Up-regulated	PITPNM2	5.281859191	0.04644272249
ENSG00000091009	Up-regulated	RBM27	5.717268862	0.04578895771
ENSG00000096654	Up-regulated	ZNF184	6.105096487	0.02190211272
ENSG00000099139	Up-regulated	PCSK5	5.617445788	0.03613978795
ENSG00000099953	Up-regulated	MMP11	6.701851739	0.0418880173
ENSG00000100100	Up-regulated	PIK3IP1	4.437388304	0.0325930285
ENSG00000100554	Up-regulated	ATP6V1D	5.383446852	0.04251892116
ENSG00000100568	Up-regulated	VTI1B	3.939601146	0.04328130598
ENSG00000100814	Up-regulated	CCNB1IP1	5.868456423	0.0255556348
ENSG00000100911	Up-regulated	PSME2	4.156122282	0.04034567367
ENSG00000100979	Up-regulated	PLTP	6.188438847	0.01155579779
ENSG00000101407	Up-regulated	TTI1	5.767856514	0.0158821791
ENSG00000101986	Up-regulated	ABCD1	5.262402957	0.03528115157
ENSG00000102030	Up-regulated	NAA10	6.100388926	0.01337110367
ENSG00000102878	Up-regulated	HSF4	5.879621467	0.04644272249
ENSG00000102934	Up-regulated	PLLP	5.440635241	0.03981019399
ENSG00000103035	Up-regulated	PSMD7	6.100108894	0.01060624479
ENSG00000103047	Up-regulated	TANGO6	5.846044076	0.02556494631
ENSG00000103489	Up-regulated	XYLT1	6.552414457	0.006920683522
ENSG00000103544	Up-regulated	VPS35L	6.763501299	0.003033710542
ENSG00000103852	Up-regulated	TTC23	6.809019664	0.008441629126
ENSG00000104731	Up-regulated	KLHDC4	5.615797394	0.008441629126
ENSG00000104856	Up-regulated	RELB	5.38373809	0.02916391327
ENSG00000104880	Up-regulated	ARHGEF18	6.016696463	0.01547513793
ENSG00000105974	Up-regulated	CAV1	5.682641711	0.01337110367
ENSG00000106524	Up-regulated	ANKMY2	5.710302462	0.0325930285

ENSG00000107745	Up-regulated	MICU1	5.167470818	0.01400675526
ENSG00000108953	Up-regulated	YWHAE	5.244021341	0.01337110367
ENSG00000112182	Up-regulated	BACH2	5.632023312	0.04167600417
ENSG00000112559	Up-regulated	MDFI	6.044024075	0.04481179636
ENSG00000112562	Up-regulated	SMOC2	6.365674938	0.01839860745
ENSG00000117222	Up-regulated	RBBP5	4.303661098	0.04586522791
ENSG00000119203	Up-regulated	CPSF3	5.152667467	0.0255556348
ENSG00000120784	Up-regulated	ZFP30	5.510912318	0.01547513793
ENSG00000121064	Up-regulated	SCPEP1	5.648762993	0.02190211272
ENSG00000121297	Up-regulated	TSHZ3	4.70470256	0.02190211272
ENSG00000121413	Up-regulated	ZSCAN18	6.436717246	0.006920683522
ENSG00000126709	Up-regulated	IFI6	5.763214556	0.01155579779
ENSG00000127329	Up-regulated	PTPRB	4.963741105	0.02404314784
ENSG00000129946	Up-regulated	SHC2	5.951807223	0.01337110367
ENSG00000130479	Up-regulated	MAP1S	5.640643851	0.0420955373
ENSG00000130638	Up-regulated	ATXN10	4.924866546	0.04353276464
ENSG00000130830	Up-regulated	MPP1	5.963870525	0.02190326915
ENSG00000132205	Up-regulated	EMILIN2	6.055849123	0.01360135701
ENSG00000132613	Up-regulated	MTSS2	4.789376868	0.0325930285
ENSG00000132854	Up-regulated	KANK4	5.841819091	0.02190211272
ENSG00000133065	Up-regulated	SLC41A1	5.714014267	0.0255556348
ENSG00000133216	Up-regulated	EPHB2	5.084178508	0.04592712441
ENSG00000135318	Up-regulated	NT5E	5.912514742	0.01800653006
ENSG00000135482	Up-regulated	ZC3H10	6.190262278	0.01782768517
ENSG00000136280	Up-regulated	CCM2	5.55708437	0.02190211272
ENSG00000138642	Up-regulated	HERC6	6.39239115	0.006920683522
ENSG00000139567	Up-regulated	ACVRL1	6.283760665	0.01547513793
ENSG00000139910	Up-regulated	NOVA1	6.111099606	0.0255556348
ENSG00000140450	Up-regulated	ARRDC4	5.802845572	0.02848811617
ENSG00000142227	Up-regulated	EMP3	5.666392061	0.02448621319
ENSG00000142675	Up-regulated	CNKSR1	5.529698551	0.02655806747
ENSG00000142875	Up-regulated	PRKACB	6.289118905	0.03668119484
ENSG00000143226	Up-regulated	FCGR2A	5.860411948	0.01337110367
ENSG00000143226	Up-regulated	FCGR2C	5.860411948	0.01337110367
ENSG00000143862	Up-regulated	ARL8A	4.161333082	0.04167600417
ENSG00000143867	Up-regulated	OSR1	7.46035064	0.01337110367
ENSG00000144043	Up-regulated	TEX261	6.464745579	0.008441629126
ENSG00000145623	Up-regulated	OSMR	6.782996929	0.003033710542
ENSG00000146592	Up-regulated	CREB5	5.700324051	0.01839860745
ENSG00000146648	Up-regulated	EGFR	4.054570519	0.04126247639
ENSG00000147408	Up-regulated	CSGALNACT1	5.882704002	0.01547513793
ENSG00000149596	Up-regulated	JPH2	6.150202579	0.02448621319
ENSG00000153291	Up-regulated	SLC25A27	6.712764891	0.02921003357
ENSG00000154874	Up-regulated	CCDC144B	6.22580065	0.02893613778
ENSG00000155090	Up-regulated	KLF10	4.952632556	0.01547513793
ENSG00000157168	Up-regulated	NRG1	6.595761685	0.01337110367
ENSG00000160111	Up-regulated	CPAMD8	5.280402261	0.04578895771
ENSG00000160255	Up-regulated	ITGB2	6.759091994	0.003033710542
ENSG00000160336	Up-regulated	ZNF761	4.963677638	0.02371491024
ENSG00000160336	Up-regulated	ZNF765-ZNF761	4.963677638	0.02371491024

ENSG00000163083	Up-regulated	INHBB	5.988786441	0.03606118789
ENSG00000164347	Up-regulated	GFM2	5.21987406	0.01800653006
ENSG00000165030	Up-regulated	NFIL3	5.596873548	0.03017023031
ENSG00000165632	Up-regulated	TAF3	5.654517418	0.01773748569
ENSG00000166266	Up-regulated	CUL5	6.374394979	0.02190211272
ENSG00000166398	Up-regulated	KIAA0355	5.162728097	0.02893613778
ENSG00000166482	Up-regulated	MFAP4	5.382978084	0.02893613778
ENSG00000166783	Up-regulated	MARF1	5.195106169	0.04671766501
ENSG00000167767	Up-regulated	KRT80	6.281922087	0.02916391327
ENSG00000168994	Up-regulated	PXDC1	5.544586566	0.01782768517
ENSG00000170264	Up-regulated	FAM161A	5.448894625	0.04578895771
ENSG00000170542	Up-regulated	SERPINB9	5.341802833	0.04076683904
ENSG00000170561	Up-regulated	IRX2	6.686655014	0.02123340818
ENSG00000170962	Up-regulated	PDGFD	5.722206319	0.03655346594
ENSG00000172201	Up-regulated	ID4	6.641175197	0.01337110367
ENSG00000172716	Up-regulated	SLFN11	5.747624758	0.02190326915
ENSG00000172889	Up-regulated	EGFL7	6.038760148	0.01337110367
ENSG00000172893	Up-regulated	DHCR7	6.231393043	0.04049189821
ENSG00000172985	Up-regulated	SH3RF3	6.092968605	0.04167600417
ENSG00000173040	Up-regulated	EVC2	5.74113444	0.02012426083
ENSG00000174348	Up-regulated	PODN	5.461166718	0.01165244135
ENSG00000175792	Up-regulated	RUVBL1	5.386320496	0.02893613778
ENSG00000176435	Up-regulated	CLEC14A	5.359882604	0.03965293199
ENSG00000177374	Up-regulated	HIC1	6.139909952	0.01337110367
ENSG00000177469	Up-regulated	CAVIN1	7.088982253	0.0002672655466
ENSG00000177613	Up-regulated	CSTF2T	5.980602079	0.02921003357
ENSG00000177706	Up-regulated	FAM20C	6.231361102	0.0255556348
ENSG00000177873	Up-regulated	ZNF619	5.080645519	0.04167600417
ENSG00000178105	Up-regulated	DDX10	5.865977647	0.04269984031
ENSG00000178700	Up-regulated	DHFR2	5.428164248	0.02921003357
ENSG00000179950	Up-regulated	PUF60	6.141961057	0.04142432355
ENSG00000179954	Up-regulated	SSC5D	4.589092654	0.04167600417
ENSG00000181019	Up-regulated	NQO1	4.565476036	0.03613978795
ENSG00000181638	Up-regulated	ZFP41	5.427627393	0.0418605097
ENSG00000181873	Up-regulated	IBA57	4.320962341	0.04592712441
ENSG00000182175	Up-regulated	RGMA	5.32934948	0.04644272249
ENSG00000188313	Up-regulated	PLSCR1	6.31692974	0.008518401272
ENSG00000188906	Up-regulated	LRRK2	6.292627116	0.01337110367
ENSG00000196155	Up-regulated	PLEKHG4	5.108135043	0.0394716171
ENSG00000196352	Up-regulated	CD55	4.155271793	0.0255556348
ENSG00000196459	Up-regulated	TRAPPC2	5.73863337	0.01719213216
ENSG00000196683	Up-regulated	TOMM7	6.050434142	0.01337110367
ENSG00000196696	Up-regulated	PDXDC2P-NPIPB14P	5.728919897	0.03118651842
ENSG00000196757	Up-regulated	ZNF700	4.990194955	0.04576666031
ENSG00000196878	Up-regulated	LAMB3	7.928843062	0.009116720707
ENSG00000197044	Up-regulated	ZNF441	5.753890848	0.02448621319
ENSG00000197128	Up-regulated	ZNF772	6.334146065	0.01337110367
ENSG00000197442	Up-regulated	MAP3K5	5.161249433	0.03374671408
ENSG00000197852	Up-regulated	INKA2	5.08712648	0.04578895771
ENSG00000198753	Up-regulated	PLXNB3	6.062880079	0.03655346594

ENSG00000198832	Up-regulated	SELENOM	6.266569173	0.01782768517
ENSG00000198912	Up-regulated	C1orf174	6.168082528	0.02916391327
ENSG00000203326	Up-regulated	ZNF525	6.251417952	0.0325930285
ENSG00000203392	Up-regulated	NA	5.612465756	0.02893613778
ENSG00000204304	Up-regulated	PBX2	5.261344975	0.04481179636
ENSG00000204764	Up-regulated	RANBP17	6.026873858	0.01096218206
ENSG00000205213	Up-regulated	LGR4	6.369065936	0.02655806747
ENSG00000206418	Up-regulated	RAB12	6.046283429	0.01060624479
ENSG00000213551	Up-regulated	DNAJC9	5.752034807	0.02437245621
ENSG00000214135	Up-regulated	LOC220729	5.248607811	0.04076683904
ENSG00000215559	Up-regulated	ANKRD20A11P	6.295080123	0.03374671408
ENSG00000215788	Up-regulated	TNFRSF25	7.041361449	0.003033710542
ENSG00000221823	Up-regulated	PPP3R1	5.959103075	0.04020457039
ENSG00000223705	Up-regulated	NSUN5P1	6.565020545	0.01337110367
ENSG00000223768	Up-regulated	LINC00205	4.319470715	0.03613978795
ENSG00000234608	Up-regulated	MAPKAPK5-AS1	6.301002004	0.01337110367
ENSG00000240038	Up-regulated	AMY2B	5.97988179	0.04921383394
ENSG00000240184	Up-regulated	PCDHGC3	5.780877493	0.02448621319
ENSG00000242588	Up-regulated	NA	5.705356928	0.02535231463
ENSG00000243302	Up-regulated	NA	6.303529672	0.009116720707
ENSG00000248994	Up-regulated	NA	6.206127216	0.02893613778
ENSG00000253731	Up-regulated	PCDHGA6	5.735241761	0.03501389341
ENSG00000255112	Up-regulated	CHMP1B	5.457100669	0.01547513793
ENSG00000256235	Up-regulated	SMIM3	5.604569194	0.03725082436
ENSG00000259291	Up-regulated	ZNF710-AS1	5.058869961	0.04049189821
ENSG00000259583	Up-regulated	LOC101927751	5.706820182	0.02893613778
ENSG00000260314	Up-regulated	MRC1	5.495862295	0.03653797719
ENSG00000271643	Up-regulated	NA	5.416104716	0.04251892116
ENSG00000272746	Up-regulated	NA	6.018746102	0.02190211272
ENSG00000285106	Up-regulated	NA	5.909911554	0.03324545981
ENSG00000286619	Up-regulated	NA	6.256269598	0.0378479853
ENSG00000287736	Up-regulated	NA	5.241554416	0.04356974883
