# Supplementary material for: Human Endogenous Retrovirus Expression Is Upregulated in the Breast Cancer Microenvironment of HIV Infected Women: A Pilot Study
Source: Front Oncol. 2020 Oct 22;10:553983. doi: 10.3389/fonc.2020.553983 (PMC7649802; doi:10.3389/fonc.2020.553983)
Supplement: Supplementary file 3 [file Data_Sheet_3.PDF]

Table S3 - GSEA result with p-value and false discovery rate (FDR)

Pathways	User ID	Gene Symbol	Gene Name	p-value	FDR
Gene expression (Transcription)	ENSG00000018408	WWTR1	WW domain containing transcription regulator 1	0.3873	1
	ENSG00000082014	SMARCD3	SWI/SNF related, matrix associated, actin dependent regulator of chromatin, subfamily d, member 3		
	ENSG00000100911	PSME2	proteasome activator subunit 2		
	ENSG00000108953	YWHAE	tyrosine 3-monooxygenase/tryptophan 5-monooxygenase activation protein epsilon		
	ENSG00000117222	RBBP5	RB binding protein 5, histone lysine methyltransferase complex subunit		
	ENSG00000119203	CPSF3	cleavage and polyadenylation specific factor 3		
	ENSG00000146648	EGFR	epidermal growth factor receptor		
	ENSG00000160336	ZNF761	zinc finger protein 761		
	ENSG00000177873	ZNF619	zinc finger protein 619		
	ENSG00000196757	ZNF700	zinc finger protein 700		
PIP3 activates AKT signaling	ENSG00000070770	CSNK2A2	casein kinase 2 alpha 2	0.33165	1
	ENSG00000103035	PSMD7	proteasome 26S subunit, non-ATPase 7		
	ENSG00000157168	NRG1	neuregulin 1		
Membrane Trafficking	ENSG00000008256	CYTH3	cytohesin 3	0.2399	1
	ENSG00000108953	YWHAE	tyrosine 3-monooxygenase/tryptophan 5-monooxygenase activation protein epsilon		
	ENSG00000146648	EGFR	epidermal growth factor receptor		
	ENSG00000196352	CD55	CD55 molecule (Cromer blood group)		
Extracellular matrix organization	ENSG00000099953	MMP11	matrix metallopeptidase 11	0.021818	0.86859
	ENSG00000160255	ITGB2	integrin subunit beta 2		
	ENSG00000196878	LAMB3	laminin subunit beta 3		
Metabolism of lipids	ENSG00000070770	CSNK2A2	casein kinase 2 alpha 2	0.68697	0.95617
	ENSG00000075239	ACAT1	acetyl-CoA acetyltransferase 1		
	ENSG00000082014	SMARCD3	SWI/SNF related, matrix associated, actin dependent regulator of chromatin, subfamily d, member 3		
	ENSG00000090975	PITPNM2	phosphatidylinositol transfer protein membrane associated 2		
	ENSG00000101986	ABCD1	ATP binding cassette subfamily D member 1		
	ENSG00000105974	CAV1	caveolin 1		
	ENSG00000142875	PRKACB	protein kinase cAMP-activated catalytic subunit beta		
	ENSG00000172893	DHCR7	7-dehydrocholesterol reductase		
Immune System	ENSG00000103544	VPS35L	VPS35 endosomal protein sorting factor like	0.19971	0.8517
	ENSG00000138642	HERC6	HECT and RLD domain containing E3 ubiquitin protein ligase family member 6		
	ENSG00000142875	PRKACB	protein kinase cAMP-activated catalytic subunit beta		
	ENSG00000145623	OSMR	oncostatin M receptor		
	ENSG00000160255	ITGB2	integrin subunit beta 2		
	ENSG00000166266	CUL5	cullin 5		
	ENSG00000215788	TNFRSF25	TNF receptor superfamily member 25		
