# Supplementary material for: Human Endogenous Retrovirus Expression Is Upregulated in the Breast Cancer Microenvironment of HIV Infected Women: A Pilot Study
Source: Front Oncol. 2020 Oct 22;10:553983. doi: 10.3389/fonc.2020.553983 (PMC7649802; doi:10.3389/fonc.2020.553983)
Supplement: Supplementary file 4 [file Data_Sheet_4.pdf]

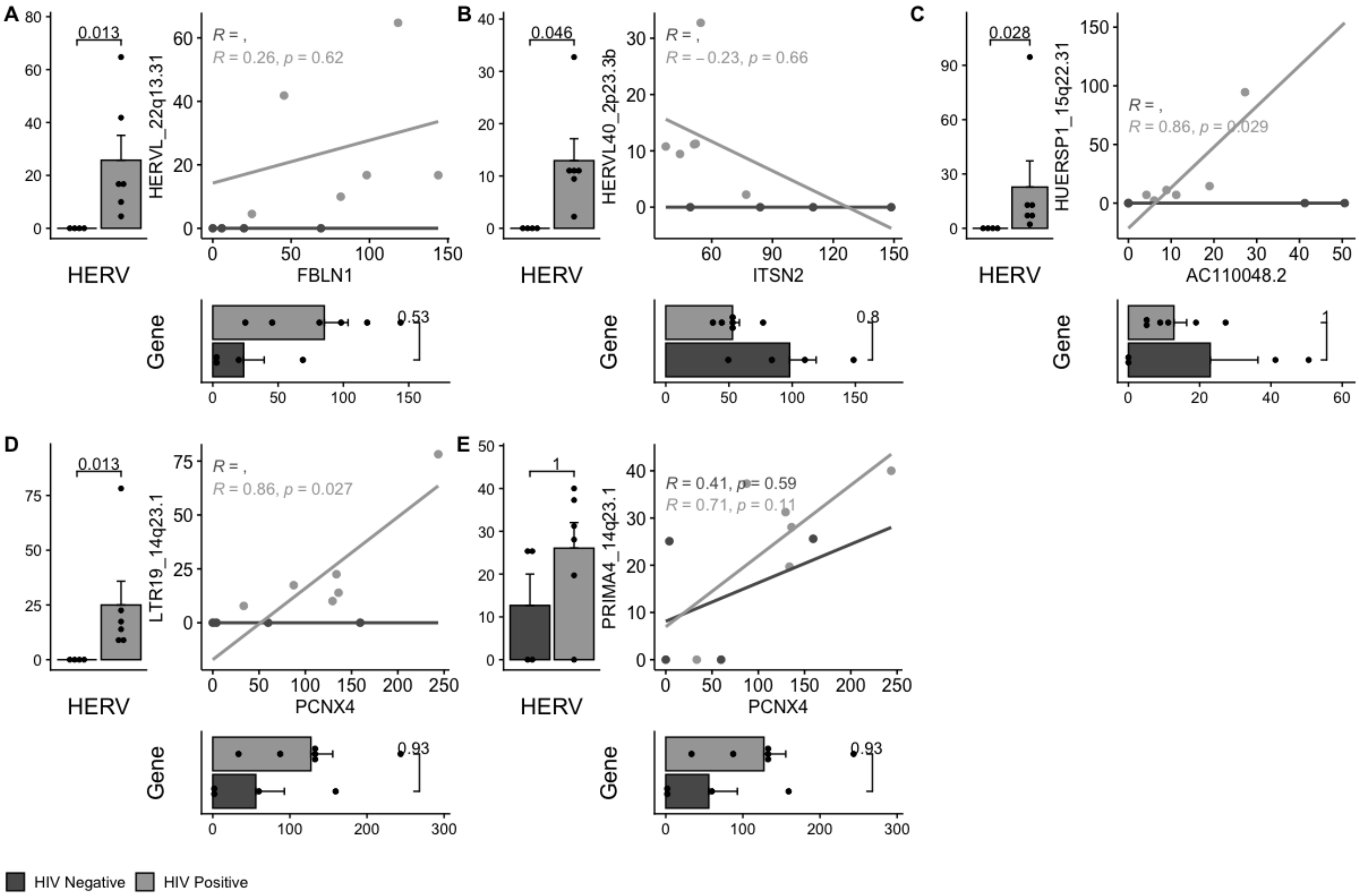

Supplementary Figure 1. Expression of host genes to nearby differentially expressed HERVs. The bar plots show the normalized HERVL_22q13.31 (A), HERVL40_2p23.3b (B), HUERSP1_15q22.31, LTR19_14q23.1 (C), LTR19_14q23.1 (D), PRIMA_14q23.1 (E) and normalized *FBLN1* (A), *ITSN2* (B), *AC110048.2* (C), *PCNX4* (D and E) gene expression between HIV-positive and HIV-negative samples. The adjusted p-value is shown in each case. Linear regression plots show HERVs and their neighbor gene expression, including the correlation coefficient (R) and the p-value.

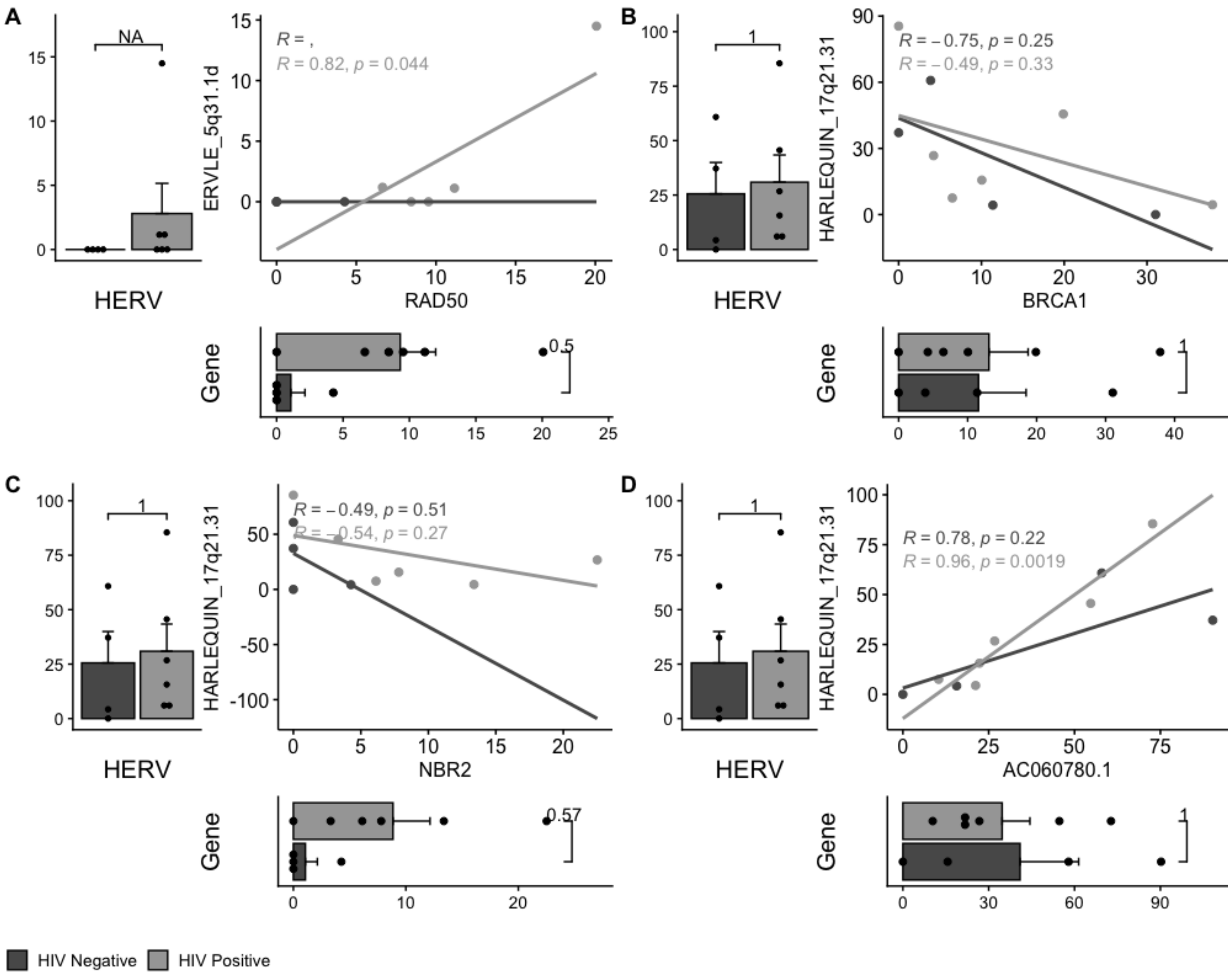

Supplementary Figure 2. Expression of breast cancer oncogenes with nearby HERVs. The bar plots show the normalized ERVLE_5q31.1d (A), HARLERQUIN_17q21.31 (B, C and D) and normalized *RAD50* (A), *BRCA1* (B), *NBR2* (C), *AC060780.1* (D) gene expression between HIV-positive and HIV-negative samples. The adjusted p-value is shown. Linear regression plots show HERVs and its neighbor gene expression, including the correlation coefficient (R) and the p-value.

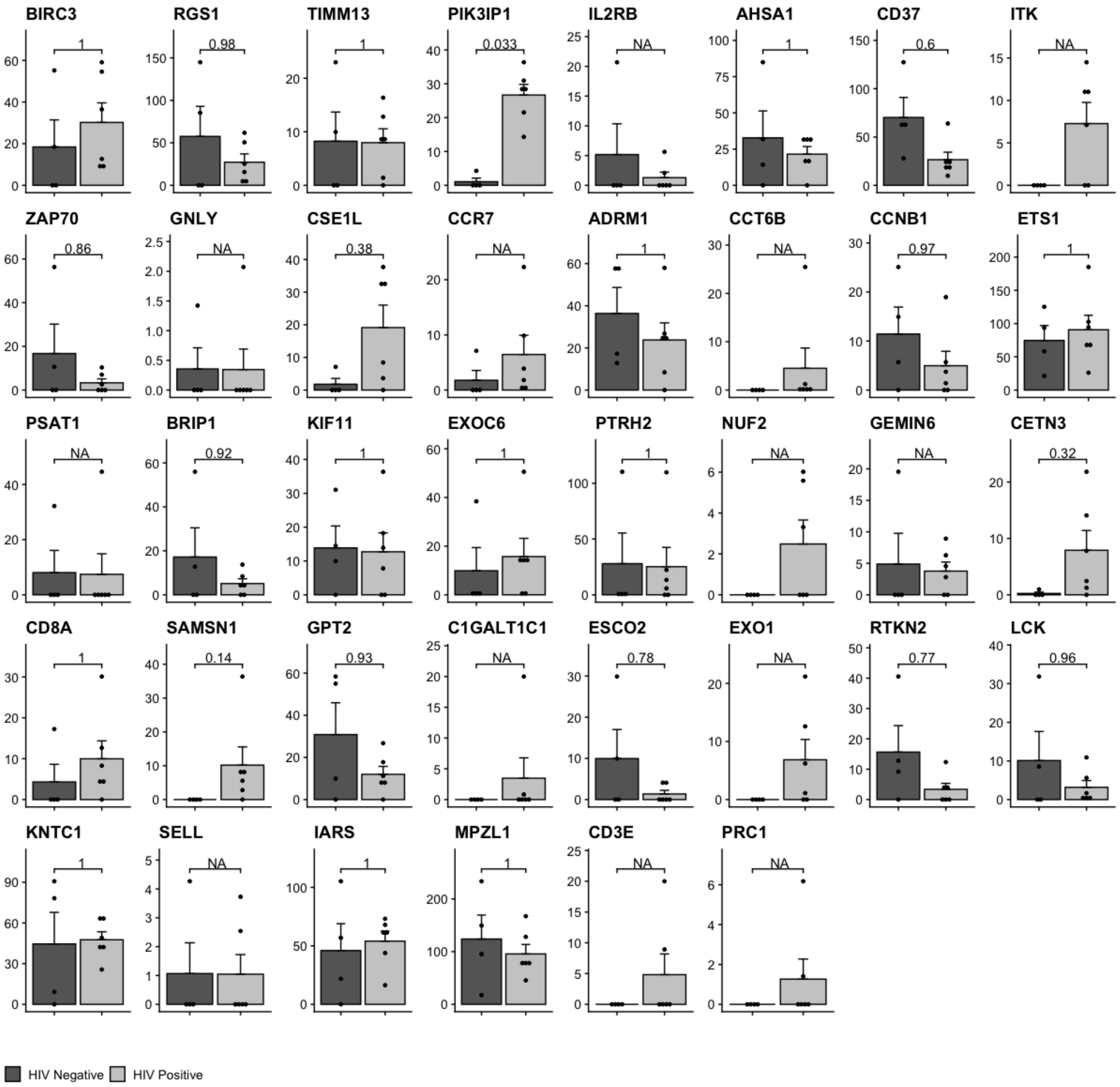

Supplementary Figure 3. Immune T-cell signature gene expression in HIV-positive and HIV-negative samples. The adjusted p-value is shown. NA, not available values due to exceedingly low gene expression according to DESeq2.
